# Risk of Cardiovascular Events in People with HIV (PWH) Treated with Integrase Strand-Transfer Inhibitors: The Debate Is Not Over; Results of the SCOLTA Study

**DOI:** 10.3390/v16040613

**Published:** 2024-04-15

**Authors:** Nicolò Corti, Barbara Menzaghi, Giancarlo Orofino, Marta Guastavigna, Filippo Lagi, Antonio Di Biagio, Lucia Taramasso, Giuseppe Vittorio De Socio, Chiara Molteni, Giordano Madeddu, Elena Salomoni, Giovanni Francesco Pellicanò, Emanuele Pontali, Rita Bellagamba, Benedetto Maurizio Celesia, Antonio Cascio, Eleonora Sarchi, Roberto Gulminetti, Leonardo Calza, Paolo Maggi, Giovanni Cenderello, Alessandra Bandera, Maria Aurora Carleo, Katia Falasca, Sergio Ferrara, Salvatore Martini, Giuliana Guadagnino, Goffredo Angioni, Olivia Bargiacchi, Elena Delfina Ricci, Nicola Squillace, Paolo Bonfanti

**Affiliations:** 1 Infectious Diseases Unit, Fondazione IRCCS San Gerardo dei Tintori, 20900 Monza, Italy; n.corti7@campus.unimib.it (N.C.); nicola.squillace@irccs-sangerardo.it (N.S.); paolo.bonfanti@unimib.it (P.B.); 2 School of Medicine and Surgery, University of Milano-Bicocca, 20900 Monza, Italy; 3 Unit of Infectious Diseases, ASST della Valle Olona, 21052 Busto Arsizio, Italy; barbara.menzaghi@asst-valleolona.it; 4 Division I of Infectious and Tropical Diseases, ASL Città di Torino, 10149 Turin, Italy; giancarlo.orofino@aslcittaditorino.it (G.O.); martaguastavigna@gmail.com (M.G.); 5 AOU Infectious and Tropical Diseases, Careggi Hospital, 50134 Florence, Italy; lagif@aou-careggi.toscana.it; 6 Infectious Diseases Clinic, IRCCS Ospedale Policlinico San Martino, 16132 Genoa, Italy; antonio.dibiagio@hsanmartino.it (A.D.B.); taramasso.lucia@gmail.com (L.T.); 7 Department of Health’s Sciences (DISSAL), University of Genoa, 16132 Genoa, Italy; 8 Unit of Infectious Diseases, Santa Maria Hospital, 06129 Perugia, Italy; giuseppe.desocio@ospedale.perugia.it; 9 Unit of Infectious Diseases, A. Manzoni Hospital, 23900 Lecco, Italy; c.molteni@asst-lecco.it; 10 Unit of Infectious Diseases, Department of Medicine, Surgery and Pharmacy, University of Sassari, 07100 Sassari, Italy; giordano@uniss.it; 11 Unit of Infectious Diseases, Santa Maria Annunziata Hospital, 50012 Florence, Italy; elena.salomoni@uslcentro.toscana.it; 12 Unit of Infectious Diseases, Department of Human Pathology of the Adult and the Developmental Age “G. Barresi”, University of Messina, 98125 Messina, Italy; gpellicano@unime.it; 13 Department of Infectious Diseases, Galliera Hospital, 16128 Genoa, Italy; emanuele.pontali@galliera.it; 14 National Institute for Infectious Diseases, Lazzaro Spallanzani Institute for Hospitalization and Care Scientific, 00149 Rome, Italy; rita.bellagamba@inmi.it; 15 Unit of Infectious Diseases, Garibaldi Hospital, 95123 Catania, Italy; bmcelesia@gmail.com; 16 Unit of Infectious Diseases, Department of Health Promotion, Mother and Child Care, Internal Medicine and Medical Specialties, University of Palermo, 90127 Palermo, Italy; antonio.cascio03@unipa.it; 17 Infectious Diseases Unit, S. Antonio e Biagio e Cesare Arrigo Hospital, 15121 Alessandria, Italy; eleonora.sarchi@ospedale.al.it; 18 Fondazione IRCCS Policlinico S. Matteo, Infectious Diseases, University of Pavia, 27100 Pavia, Italy; r.gulminetti@smatteo.pv.it; 19 Department of Medical and Surgical Sciences, Clinics of Infectious Diseases, S. Orsola-Malpighi Hospital, “Alma Mater Studiorum”, University of Bologna, 40128 Bologna, Italy; leonardo.calza@unibo.it; 20 Infectious Diseases Unit, AORN Sant’Anna e San Sebastiano, 81100 Caserta, Italy; paolo.maggi@unicampania.it; 21 Infectious Diseases Department, Sanremo Hospital, 18038 Sanremo, Italy; g.cenderello@asl1.liguria.it; 22 Infectious Disease Unit, Fondazione IRCCS Ca’ Granda Ospedale Maggiore Policlinico, 20122 Milan, Italy; alessandra.bandera@unimi.it; 23 Infectious Diseases and Gender Medicine Unit, Cotugno Hospital, AO dei Colli, 80131 Naples, Italy; mariaaurora.carleo@ospedalideicolli.it; 24 Clinic of Infectious Diseases, Department of Medicine and Science of Aging, G. D’Annunzio University, Chieti-Pescara, 66100 Chieti, Italy; k.falasca@unich.it; 25 Unit of Infectious Diseases, Department of Clinical and Experimental Medicine, University of Foggia, 71122 Foggia, Italy; sferrara@ospedaliriunitifoggia.it; 26 Infectious Disease Unit, University Hospital Luigi Vanvitelli, 80138 Naples, Italy; salvatoremartini76@gmail.com; 27 Department of Infectious and Tropical Diseases, St. Annunziata Hospital, 87100 Cosenza, Italy; giuliana.guadagnino@gmail.com; 28 Infectious Diseases Unit, SS Trinità Hospital, 09121 Cagliari, Italy; goffredoangioni@gmail.com; 29 Unit of Infectious Diseases, Ospedale Maggiore della Carità, 28100 Novara, Italy; olivia.bargiacchi@gmail.com; 30 Fondazione A.S.I.A. Onlus, 20090 Buccinasco, Italy

**Keywords:** HIV, cardiovascular disease, integrase strand transfer inhibitors, observational study

## Abstract

Cardiovascular disease (CVD) is common in people with HIV (PWH), and has great impact in terms of morbidity and mortality. Several intertwined mechanisms are believed to play a role in determining the increased risk of CVD, including the effect of certain antiretrovirals; among these, the role of integrase strand-transfer inhibitors (INSTIs) is yet to be fully elucidated. We conducted a multicenter, observational study comprising 4984 PWH evaluating the antiretroviral therapy (ART)-related nature of CVD in real life settings, both in naïve vs. treatment-experienced people. A comparison was conducted between INSTIs vs. either protease inhibitors (PIs) or non-nucleoside reverse transcriptase inhibitors (NNRTIs) considering demographic, baseline clinical characteristics, incidence of CVD in both 2-year and complete follow-up periods. Among 2357 PWH exposed to INSTIs, 24 people experienced CVD; the corresponding figure was 12 cases out of 2599 PWH exposed to other ART classes. At univariate and multivariate analysis, a tendency towards an increased risk of CVD was observed in the 2-year follow-up period in PWH exposed to INSTIs in the absence, however, of statistical significance. These findings leave open the hypothesis that INSTIs may play a role, albeit minimal, in determining an increased risk of CVD in PWH.

## 1. Introduction

The human immunodeficiency virus (HIV) pandemic remains one of the most challenging public health problems since its beginning in the 1980s. Estimates suggest that at the end of 2022, around 630,000 people died from acquired immunodeficiency (AIDS)-related illnesses, and approximately 39 million people globally were living with HIV [1]. Notwithstanding, the burden and mortality of the disease have massively declined since the early 2000s, with current global HIV data even showing a clear trend towards a reduction in the number of new HIV infections per year. Alongside this important achievement, there is some important evidence that the life expectancy of people with HIV (PWH) is moving closer to that of the general population [2]. This is positive, but increases the risk of developing age-related diseases, including cardiovascular diseases (CVD).

According to certain modelling studies, it is estimated that, by 2030, 78% of PWH will be affected by cardiovascular disease (CVD) [3]. As extensively observed in the pre-ART era and the first phases of antiretroviral development, CVD significantly impacts the overall well-being of PWH, and has a great impact in terms of morbidity and mortality [4,5,6]. In general, CVD rates are higher for PWH than for the general population; this depends on several intertwined mechanisms, such as the immune changes associated with the virus itself, the persistent activation of inflammatory pathways despite adequate viro-immunological control, the high prevalence of modifiable cardiovascular risk factors in PWH, and the use of certain antiretrovirals [7]. The extent to which each single factor associated with HIV and its management determines cardiovascular (CV) risk are unknown and yet to be fully established: understanding the complex interplay of these factors and how each of them contributes to CVD has become of paramount importance, as minimizing and preventing possible comorbidities has now become a cornerstone of modern HIV medicine [8,9].

While initially associated with inevitable progression towards AIDS, HIV infection has evolved over the years into a chronic disease, since several pharmacological options have transformed it into a clinically controllable condition [1,10]. Changing the management of the infection, the advent of ART has improved PWH’s life expectancy, bringing it closer to that of the general population [11]. In addition, ART has reduced the incidence of many comorbidities associated with HIV infection, including cardiovascular events; PWH on antiretroviral treatment with virological suppression have a lower risk of events than those either untreated or not virologically suppressed [12]. However, effective ART has not fully eliminated the increased risk of CV events, which represent an important clinical challenge [13]. Recently the REPRIEVE study reported that statin treatment significantly reduced the risk of major cardiovascular events in ART-treated PWH at low CV risk. This finding makes the phenomenon even more complex to interpret [14]. While intervention on modifiable risk factors remains imperative, much interest has been put into understanding whether specific antiretrovirals may contribute further to the risk of CVD. The use of antiretroviral therapy, in general, has been originally questioned as a possible precipitating factor for CVD; in 2003, initial results from the Data Collection on Adverse events of Anti-HIV Drugs (D:A:D) cohort suggested that a longer exposure to ART was associated with a 26% relative increase in rate of myocardial infarction (MI) per year [15]. These findings have clashed with previous studies, suggesting that episodic ART use might have been beneficial in terms of limiting certain side effects associated with its use [16]; nonetheless, this conviction was soon argued against, after it was clear that continuous ART is incomparably more favorable than episodic ART. In fact, the increased CV risk could be explained by the use of specific antiretrovirals rather than ART itself, and better viro-immunological control has been established as fundamental to reduce CV risk [12]. Therefore, a tailored approach to ART choice, along with strict compliance, remains crucial in preventing CVD [17].

With several molecules now available, the question remains as to which antiretroviral better fits an individual with CV risk factors, or which has a better tolerability profile from the cardiovascular point of view [18]. In the first years of the ART era, the use of thymidine-based analogues—such as zidovudine and stavudine—seemed a reasonable compromise to limit viral replication, despite their mitochondrial toxicity [19]. When it became clear that their use was weakly associated with viral control, but strongly linked to a plethora of side effects (for instance the development of dilative cardiomyopathy [20]), interest was shifted to more tolerable antiretrovirals. For several years, abacavir (ABC) has been a pillar backbone for effective combined ART, but its use has later been discouraged because it is associated with an increased risk of MI [19]. Similarly, protease inhibitors (such as ritonavir-booster darunavir and lopinavir) are excellent antiretrovirals with a high genetic barrier. Nonetheless, they have been associated with a higher rate of MI per year of exposure and are often linked to metabolic disturbances including ineffective lipid control [21].

A turning point came with the advent of integrase strand transfer inhibitors (INSTIs), characterized by an excellent tolerability profile with scarce side effects. Initially, little evidence suggested that INSTIs may contribute to CVD [22,23]. The ADVANCE trial, an open label randomized clinical trial conducted in South Africa, demonstrated an association between dolutegravir exposure and weight gain [24]. More recently, the RESPOND cohort consortium, a prospective, multicenter collaboration study on cardiovascular events during INSTI exposure, suggested that INSTI initiation is associated with an early onset of CVD in the first two years of drug exposure [25]. These findings opened the question as to whether an INSTI-based regimen can be considered safe from the cardiovascular point of view [26,27,28]. As more recent studies seem to confirm the tolerability of INSTIs, the findings from these studies should be interpreted with caution; exploring whether INSTIs, currently the most prescribed antiretrovirals, play a role in increasing CV risk for PWH is still an open question that demands elucidations [29].

To add information on this issue, we report the results of the Surveillance Cohort Long-term Toxicity Antiretrovirals (SCOLTA) Project.

## 2. Materials and Methods

The SCOLTA project is a multicenter observational study that started in 2002 and prospectively follows PWH who start a treatment with new antiretroviral drugs to identify toxicities and adverse events (AEs) in a real-life setting [30], involving 25 Italian Infectious Disease Centers. Data are collected using an online pharmacovigilance program (www.cisai.it (accessed on 11 April 2024)), where a cohort opens for each newly marketed drug.

All PWH followed in the participating centers are eligible for the study if they are prescribed the cohort drug for the first time. As this is an observational study, the choice of therapy is entirely up to the individual physicians and PWH in each center. Both ART naïve and ART experienced PWH can be included, if they are >18 years and agree on study entry. Since 2002, cohorts for lopinavir, atazanavir, darunavir, raltegravir, rilpivirine, elvitegravir, dolutegravir, bictegravir and doravirine were enrolled and followed-up with prospectively. At study entry, information is collected about sex, age, ethnicity, weight, height, CDC stage, comorbidities, pharmacological treatments other than ART, and previous ART history. At baseline and at each six-monthly follow-up visit, laboratory data (HIV-RNA, CD4 +T cell count, total serum cholesterol, low-density lipoprotein cholesterol, high-density lipoprotein cholesterol, glycemia, triglycerides) are prospectively collected in anonymous form in a central database. Information on comorbidities and pharmacological treatments is also updated at each follow-up visit.

We recorded data regarding independent risk factors for CVD, such as hypertension, dyslipidemia, chronic kidney diseases (CKD), and diabetes. Hypertension was defined as office systolic BP of ≥140 mmHg and/or a diastolic BP of ≥90 mmHg or receiving antihypertensive therapy at the time of the examination. Dyslipidemia referred to levels of one or more kinds of lipids in the blood (triglycerides > 150 mg/dL, cholesterol > 200 mg/dL, LDL > 115 mg/dL) and/or the use of statins and other lipid-lowering drugs. CKD diagnosis was based on an estimated glomerular filtration rate < 60 mL/min per 1.73 square meters, persisting for at least 3 months. The diagnosis of diabetes was based on standard international criteria [31].

AEs are collected prospectively as soon as they are clinically observed. Patients are followed up with at 6-month intervals. Those who do not present to the physician in ≥6 months leave the study and are considered lost to follow-up. If a person stops using the cohort drug, she or he leaves the study. Severe adverse reactions or unexpected events appearing within 6 months of leaving the study are to be recorded. Events are classified for severity using the Common Terminology Criteria for Adverse Events (CTCAE) [32] (grade III and IV reactions, and all unexpected events, i.e., those not mentioned in the package insert of the drug or not reported in the clinical trials on which registration was based). The reason for cohort drug interruption is provided in the follow-up form if it is something other than an adverse event (such as treatment failure, patient’s choice, simplification, pregnancy, loss to follow-up).

PWH are observed until they interrupt the cohort drug, or the cohort closes, whichever occurs first.

In this analysis, we merged the data of nine different cohorts of the SCOLTA project: lopinavir (LPV) (2002–2006), atazanavir (ATV) (2003–2008), darunavir/ritonavir (DRV/r) or cobicistat (DRV/c) (2006–2019), rilpivirine (RPV) (2013–2017), raltegravir (RAL) (2007–2014), elvitegravir/c (EVG) (2014–2019), dolutegravir (DTG) (2014-ongoing), bictegravir (BIC) (2019-ongoing), and doravirine (DOR) (2020-ongoing) cohorts.

The original study protocol was approved on 18 September 2002, and three amendments were approved on 13 June 2013, 20 December 2019, and 12 May 2020 by the coordinating center at Hospital “L. Sacco”-University of Milan, Milan (Italy), and thereafter by all participating centers. Written consent for study participation was obtained from all participants, and the study was conducted in accordance with the ethical standards laid down in the 1964 Declaration of Helsinki and its later amendments and by Italian national laws.

### Statistical Analysis

Data were described using mean and standard deviation (SD) for normally distributed continuous variables, median, and interquartile range (IQR) for not normally distributed continuous variables, and frequency (%) for categorical and ordinal variables. Distribution normality was assessed using the graphical quantile–quantile method. Baseline differences were tested using the analysis of variance for means, the non-parametric Mann–Whitney test for medians, and proportion comparisons were performed using the chi-square test.

Since the regimens could also include ART drugs from several classes, the main comparison was performed between people who took an INSTI, regardless of the enrolment cohort, and those who did not.

Incidence rates (IR) were calculated as the number of the first CV event occurrence per 1000 person years of follow-up (PYFU), with 95% confidence intervals (CI). We also computed the rate ratio (RR and corresponding 95% CI) as the ratio of the incidence rate in the exposed group divided by the incidence rate in the unexposed comparison group. Crude and adjusted IR and RR were estimated through a generalized linear model, with the selection of a log-link Poisson function. In the multivariate model, we tested the variables that were significantly different between exposure groups and selected as potential confounders those that were significantly associated with the outcome at the univariate analysis.

To account for the different observation time among ART classes, we repeated these analyses truncating the observation at 2-year follow-up.

All *p*-values were two-sided, at the significance level < 0.05. All statistical analyses were performed using SAS for Windows 9.4 (SAS Institute, Cary, NC, USA).

## 3. Results

**Figure 1:**
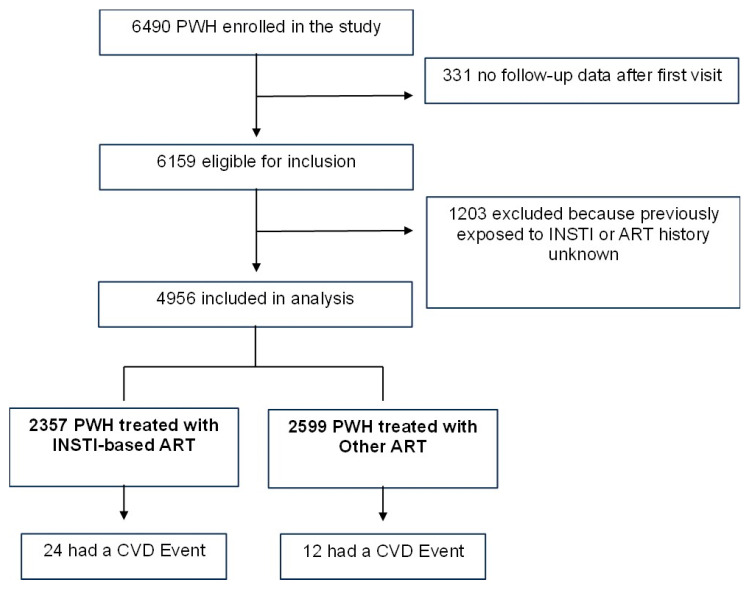
Flow chart.

Out of 6159 PWH enrolled in the SCOLTA cohorts until September 2023, with at least one follow-up visit, 1139 were excluded because they were not naïve to INSTI and 64 because their ART history could not be established: overall, 4956 PWH were included (Figure 1).

The main characteristics of PWH enrolled in the different cohorts are described in Table 1. Overall, 2357 (47.6%) PWH took a regimen including an INSTI. Comparing people starting a treatment with INSTI and those starting a PI or an NNRTI, the groups were different in terms of all variables considered, with PWH on INSTI older and less frequently having a history of intravenous drug use (IVDU). People starting an INSTI-based regimen were less frequently in CDC stage C and with CD4 + cell count < 200/mm^3^. They were also more frequently naïve to ART. On the other hand, non-infectious comorbidities were more present in this group, with about twice the frequency seen in the non-INSTI-based group for hypertension, diabetes, dyslipidemia, chronic kidney disease and previous CV events.

Overall, 38 CV events occurred: twenty-four acute myocardial infarctions (AMIs), seven ischemic strokes, three transient ischemic attack (TIA), three hemorrhagic strokes, and one sudden cardiac death. Among these PWH, two had a repeated event (one AMI in the INSTI group, one ischemic stroke in the non-INSTI group) and were thus excluded from the analysis. None of the remaining events occurred in 76 PWH with previous CVE. Out of these 36 CV events, 28 occurred in the first 2-year follow-up.

The incidence rates are reported in Table 2. Over the whole follow-up period, the crude IR was 3.1 (95% CI 2.2–4.3) events/1000 PYFU, and 3.8 (95% CI 2.6–5.5) in the first two years of follow-up.

**Figure 2:**
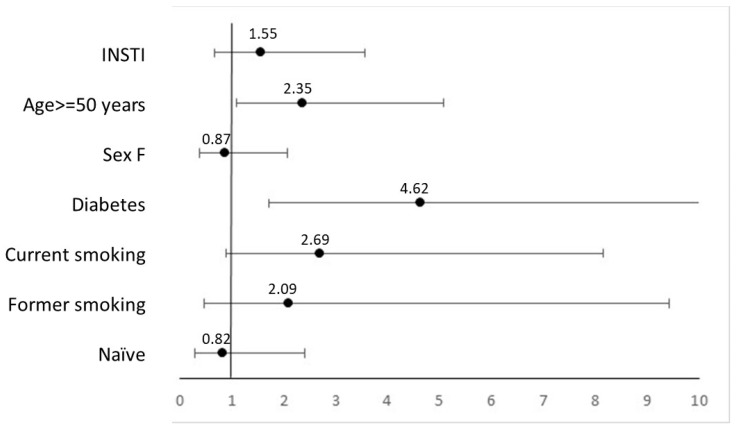
Adjusted rate ratios for cardiovascular events (2-year follow-up).

At the univariate analysis, age ≥ 50 years (crude IR 5.2, 95% CI 3.3–8.2, vs. 2.1, 95% CI 1.3–3.4, *p* = 0.007), diagnosis of diabetes (crude IR 12.1, 95% CI 5.0–29.0, vs. 2.8, 95% CI 1.9–3.9, *p* = 0.002) and current (crude IR 4.7, 95% CI 3.0–7.4, *p* = 0.02) and former (crude IR 6.2, 95% CI 2.6–14.8, *p* = 0.02) smoking, as compared to never smoking (crude IR 1.3, 95% CI 0.5–3.5) were predictors of CV events in the whole observation period. In the two years since enrolment, these results repeated in people aged ≥50 years (crude IR 6.9, 95% CI 4.2–11.5 vs. 2.5, 95% CI 1.5–4.4, *p* = 0.008) and in those with diabetes (crude IR 20.0, 95% CI 8.3–48.1, vs. 3.3, 95% CI 2.2–4.9, *p* = 0.0002), but lost significance according to smoking habits (current smokers, crude IR 5.8, 95% CI 3.5–9.7, *p* = 0.08; former smokers, crude IR 5.8, 95% CI 1.9–18.1, *p* = 0.21) in comparison with never smokers (crude IR 1.3, 95% CI 0.9–6.0). In the multivariate models, we included these variables, plus sex as a known risk factor for CVD, to compare the CVD incidence between INSTI and non-INSTI groups, accounting for known risk factor (Figure 2).

As regards INSTI treatment and ART experience, at the univariate analysis, we did not observe any significant difference, although in the 2-year observation the crude RR for INSTI-treated PWH was 2.02 (95% CI 0.91–4.46). The adjusted RR for PWH on INSTI versus those treated with other drug classes was 1.55 (95% CI 0.67–3.56, *p* = 0.30) in the 2-year period and 1.10 (95% CI 0.53–2.29, *p* = 0.80) in the whole follow-up.

## 4. Discussion

This prospective, multicenter cohort study provides a comprehensive description of both quantitative and qualitative aspects of CVD in naïve and experienced PWH exposed to INSTI or other antiretroviral drug classes between 2002 and 2023. This study has highlighted that a tendency towards a higher incidence of CVD is apparent with INSTI exposure, but several aspects must be considered.

Notwithstanding the lack of statistical significance, our findings suggest that CVD risk was higher in the two years after INSTI initiation and lowered in the following period, which is consistent with the results of previous studies [25]. Above all, our study appears to parallel the results from the RESPOND cohort, although several considerations must be taken into account. As previously described, the RESPOND cohort has highlighted that in INSTI-treated PWH the risk of CVD doubles in the first 24 months, climaxes at 6 months, and then gradually disappears, as compared to non-INSTI-treated PHW. After this timeframe, no association has been observed. This finding remained solid even after adjusting for common CVD-associated variables, such as dyslipidemia and BMI. Moreover, a higher incidence of hypertension was observed during INSTI exposure, strengthening this association. However, the accuracy of these findings has been debated for several reasons. While the results fairly departed from previous findings that assured the safety profile of the INSTI class from the CV point of view, the authors admitted the potential for unmeasured confounding and channeling bias. Similarly, although we accounted for potentially confounding factors, we cannot exclude the impact of residual channeling bias in our study, since the seemingly more CV-friendly INSTI were more frequently prescribed to people with worse cardiovascular risk profile. The INSTI group was significantly older, with fewer cases of IVDU and a higher percentage of treatment-naïve people. This group also showed a greater incidence of classical CV risk factors and comorbidities, with more individuals affected by hypertension, diabetes, dyslipidemia, and a history of previous CVD. Thus, it is possible that they were selected to receive INSTIs rather than other antiretroviral classes because of these characteristics. Using an analysis methodology that tends to attenuate channeling bias, the Swiss HIV Cohort Study recently observed no difference in CVD risk between INSTI-based and non-INSTI-based groups in treatment-naïve PWH [33].

Considering the heterogeneity of the literature, it is mandatory to define the position of our results. Our main findings partially align with those from the RESPOND cohort: concerning other molecules, such as NNRTIs and PIs, INSTIs appear to have a higher incidence of CVD. However, the global incidence of CVD observed in our study was much lower than in other studies. Thus, it would be reasonable to hypothesize that the actual burden of CVD caused by an INSTI-based regimen in our population is somewhat negligible from a clinical perspective. On the one hand, this finding may depend on the smaller sample size of our cohort compared to other studies, but it is also fundamental to evaluate the population considered. First, the analyzed population is generally less predisposed to CVD than other populations from areas of the world with a higher incidence of these events [34,35,36]. Even without considering the HIV setting, southern Europeans are less likely to suffer from CVD than northern Americans or northern Europeans, because of intricate genetic, lifestyle, and ethical issues [37,38,39]. Therefore, an increase in CV risk would, in general, translate into a small number of CV events in any case. Moreover, although smaller than other cohorts, our sample is robust in terms of number and follow-up period; therefore, it is arguable that further enrolment may have a small impact on our results. Thus, the question arises as to whether this small incidence is linked to either the intrinsic geographic characteristics of the population or whether the risk of INSTIs is, in fact, minor.

The impact of INSTI on CVD should also be considered in light of possible confounders. The INSTI-based population in our study appeared to be older, and this may reflect the comorbid status of these patients. As these diseases are markedly associated with the ageing process, it is reasonable to presume that an older population may suffer from chronic and degenerative conditions, such as diabetes, hypertension, and other CV-associated risk factors [40,41,42]. Therefore, it may be argued that the higher incidence of these events in the INSTI-based population reflects the population sample a priori. However, when the IR and RR of CVD were adjusted for the potential confounders, a higher rate was observed both in the 2-year and complete follow-up periods. Despite the lack of statistical significance, these results suggest a slightly higher risk of CV events at the start of an INSTI-based treatment.

With INSTIs being the most prescribed antiretrovirals as suggested by international guidelines [8,9], the concern regarding CVD should be interpreted considering the aspects that have made these molecules the most tolerable since the beginning of the ART era. INSTIs are safe, efficacious, practically formulated as single-tablet regimens, and bring about a series of advantages, including their relatively lower number of side effects compared to other antiretrovirals. This is probably why their potential hazard on CV risk has not raised much concern within the medical community [29]. Moreover, at the time of writing, it is difficult to find acceptable alternatives to INSTIs when prescribing an antiretroviral regimen. While some people may display a high CV risk profile, other options may be considered. For example, doravirine, a new NNRTI, has gained prominence for its effectiveness in managing HIV while being well tolerated from a metabolic perspective [43]. Conversely, newer drug classes have limited data regarding their impact on CV health, with some only recently available (e.g., injectable long-acting agents) and some still to be approved by committees (i.e., lenacapavir, islatravir, etc.). It is fair to assume that much time will pass before data on CV risk will be provided.

Amid these concerns, it is crucial to recognize that INSTIs continue to offer substantial benefits. As a tailored approach to ART constitutes one of the cornerstones of modern HIV medicine, the key to reducing ART risks lies in individualized treatment plans. With a better understanding of the CV risk associated with INSTIs and the timeframe during which this risk manifests as a disease, a comprehensive approach may minimize the hazard. As specific guidelines on CVD in PWH suggest [7], assessing and managing common CV risk factors remain the first step for a correct CVD management. Considering HIV as a global infection that affects various organs and systems, it is essential to consider multidisciplinarity in the selection of the best regimen for each patient. This approach should address CV risk precisely when it is most likely to manifest as a disease. This way, INSTIs can continue to serve as the primary treatment option, and concurrent efforts can be made to lower other pre-existing CV risk factors, ultimately improving the overall health and well-being of PWH.

This study has several limitations to acknowledge. First, the Infectious Diseases Clinics that participated in the SCOLTA study were not selected randomly at a national level, but rather volunteered to participate observationally: because of this, our findings may not be fully representative of the Italian scenario. Furthermore, the results of this study should be limited to populations that are similar to the one analyzed. In particular, our sample only enrolled PWH that started a treatment including a newly marketed antiretroviral drug, thus selecting people in need of changing or initiating ART for any reasons (i.e., failure or toxicity of previous regimens, or new diagnosis of HIV infection). Moreover, when the individual interrupted the study drug, they were not further followed-up with. Nonetheless, our research has several notable strengths. We described a comprehensive dataset, which includes a detailed description of a large group of INSTI-treated people and a thorough overview of their characteristics in real-world settings.

## 5. Conclusions

In summary, our research suggested that an increase in CV risk may occur during INSTI exposure, particularly in the initial two years of treatment. Nevertheless, the lack of statistical significance prevents us from drawing any conclusions. The need of further research is compelling because, even if the recent findings do not tarnish the excellent pharmacological features of INSTIs, they raise the issue whether INSTIs may increase CV risk in certain people. It is essential to individualize risk assessments for each patient and incorporate CV risk factors evaluations into follow-up appointments, implementing strategies to mitigate these risks. Early intervention and tailored care may ensure the benefits of INSTIs outweigh their potential risks. As new treatment options become available, the medical community can refine its approach, providing PWH with the most effective and safe treatment regimen, ultimately promoting personalized medicine for a healthier future and improved quality of life.

## Figures and Tables

**Table 1:** Characteristics of 4956 PLWH at enrolment in the SCOLTA project, according to enrolment cohort.

Characteristics	INSTI N = 2357 (47.6%)	Non INSTI N = 2599 (52.4%)	Total N = 4956	*p*-Value *
Male sex, n (%)	1750 (74.2)	1862 (71.6)	3612 (72.9)	0.04
Age, years (median, IQR)	47.0 (40.0–54.0)	42.0 (37.0–48.0)	44.0 (38.0–51.0)	<0.0001
Caucasian ethnicity, n (%)	2140 (90.8)	2405 (92.5)	4545 (91.7)	0.03
BMI > 30.0 kg/m^2^, n (%)	161 (6.8)	62 (2.4)	223 (4.5)	0.49
missing	301 (12.8)	1725 (66.4)	2026 (40.9)	
Intravenous drug use, n (%)	501 (21.3)	879 (33.8)	1380 (27.8)	<0.0001
CDC stage C, n (%)	624 (26.5)	812 (31.2)	1436 (29.0)	0.0003
CD4 cells/mm^3^ < 200, n (%)	507 (21.5)	704 (27.1)	1211 (24.4)	<0.0001
Naïve, n (%)	636 (27.0)	384 (14.8)	1020 (20.6)	<0.0001
Hypertension, n (%)	379 (16.1)	211 (8.1)	590 (11.9)	<0.0001
Diabetes, n (%)	106 (4.5)	67 (2.6)	173 (3.5)	0.0002
Dyslipidaemia, n (%)	326 (13.8)	170 (6.5)	496 (10.0)	<0.0001
Chronic kidney disease, n (%)	32 (1.4)	15 (0.6)	47 (0.9)	0.005
Previous cardiovascular event, n (%)	54 (2.3)	24 (0.9)	78 (1.6)	0.0001
Ever smoker, n (%)	1147 (48.7)	943 (36.3)	2090 (42.2)	<0.0001
Never smoker, n (%)	740 (31.4)	415 (16.0)	1155 (23.3)	
ND, n (%)	470 (19.9)	1241 (47.7)	1711 (34.5)	

BMI: body mass index; INSTI: integrase strand transfer inhibitors; IQR: interquartile range; ND: not determined; PLWH: people living with HIV. * from analysis of variance for means, Mann–Whitney test for medians, chi-square test for proportions.

**Table 2:** Incidence rates and rate ratios of first CV events (2-year and complete follow-up).

	N	Events	PYFU	Crude IR/1000 PYFU (95% CI)	Crude RR (95% CI)	Adjusted * IR/1000 PYFU (95% CI)	Adjusted * RR (95% CI)
***2-year follow-up***							
Total	4984	28	7321.37	3.8 (2.6–5.5)		6.7 (3.6–12.2)	
INSTI	2192	19	3736.59	5.1 (3.2–8.0)	2.02 (0.91–4.46)	8.7 (4.5–16.7)	1.55 (0.67–3.56)
Non INSTI	2792	9	3574.54	2.5 (1.3–4.8)		5.6 (2.4–12.8)	
Naïve	1020	4	1459.94	2.7 (1.0–7.3)	0.67 (0.23–1.93)	6.2 (2.0–19.0)	0.82 (0.28–2.39)
Experienced	3964	24	5851.18	4.1 (2.7–6.1)		7.6 (4.1–14.0)	
***Complete follow-up***							
Total	4984	36	11,688.51	3.1 (2.2–4.3)		7.4 (4.1–14.5)	
INSTI	2356	24	6770.75	3.5 (2.4–5.3)	1.45 (0.72–2.90)	5.4 (2.9–10.0)	1.10 (0.53–2.29)
Non INSTI	2598	12	4907.51	2.4 (1.4–4.3)		4.9 (2.3–10.5)	
Naïve	1020	5	2149.58	2.3 (1.0–5.6)	0.72 (0.28–1.84)	4.8 (1.7–13.3)	0.91 (0.35–2.36)
Experienced	3934	31	9528.67	3.2 (2.3–4.6)		5.3 (3.0–9.4)	

* Including age ≥ 50 years, diabetes (Y/N), smoking habits (never, former, and current smokers; a class was added for missing information on smoking, since this data was missing in about one third of the sample). CI: confidence interval; INSTI: integrase strand transfer inhibitors; IR: incidence rate; PYFU: person year follow-up; RR: rate ratio.

## Data Availability

The data presented in this study are available on reasonable request from the corresponding author due to restrictions.
